# Supplementary material for: Retention time prediction using neural networks increases identifications in crosslinking mass spectrometry
Source: Nat Commun. 2021 May 28;12:3237. doi: 10.1038/s41467-021-23441-0 (PMC8163845; doi:10.1038/s41467-021-23441-0)
Supplement: Supplementary file 3 — Reporting Summary [file 41467_2021_23441_MOESM3_ESM.pdf]

nature research

Corresponding author(s): Juri Rappsilber

Last updated by author(s): 20/4/21

Reporting Summary

Nature Research wishes to improve the reproducibility of the work that we publish. This form provides structure for consistency and transparency in reporting. For further information on Nature Research policies, see our Editorial Policies and the Editorial Policy Checklist.

Statistics

For all statistical analyses, confirm that the following items are present in the figure legend, table legend, main text, or Methods section.

n/a	Confirmed	
☐	☒	The exact sample size (*n*) for each experimental group/condition, given as a discrete number and unit of measurement
☐	☒	A statement on whether measurements were taken from distinct samples or whether the same sample was measured repeatedly
☐	☒	The statistical test(s) used AND whether they are one- or two-sided<br>*Only common tests should be described solely by name; describe more complex techniques in the Methods section.*
☐	☒	A description of all covariates tested
☐	☒	A description of any assumptions or corrections, such as tests of normality and adjustment for multiple comparisons
☐	☒	A full description of the statistical parameters including central tendency (e.g. means) or other basic estimates (e.g. regression coefficient) AND variation (e.g. standard deviation) or associated estimates of uncertainty (e.g. confidence intervals)
☐	☒	For null hypothesis testing, the test statistic (e.g. *F*, *t*, *r*) with confidence intervals, effect sizes, degrees of freedom and *P* value noted<br>*Give P values as exact values whenever suitable.*
☒	☐	For Bayesian analysis, information on the choice of priors and Markov chain Monte Carlo settings
☒	☐	For hierarchical and complex designs, identification of the appropriate level for tests and full reporting of outcomes
☒	☐	Estimates of effect sizes (e.g. Cohen's *d*, Pearson's *r*), indicating how they were calculated

Our web collection on statistics for biologists contains articles on many of the points above.

Software and code

Policy information about availability of computer code

Data collection	Mass spectrometry data were acquired with an Q Exactive HF mass spectrometer (Thermo Fisher Scientific, Bremen, Germany, Tune 2.9.3.2948, Xcalibur 4.1.31, SII for Xcalibur 1.4) coupled to an Ultimate 3000 RSLC nano system (Dionex, Thermo Fisher Scientific, Sunnyvale, USA).
Data analysis	xiSEARCH (v. 1.6.753), xiFDR(v. 2.1.3 and 2.1.5) , Comet (v. 2019010), msConvert (3.0.20175.cbf82d022), xiRT (v. >1.1), pLink2 (v. 2.3.9), seaborn (0.11.0), SHAP (0.36.0), sklearn (0.24.1), tensorflow (1.15 and >2), python 3.8.3/3.7, xiVIEW (https://xiview.org/xiNET_website/index.php, beta). xiRT (https://github.com/Rappsilber-Laboratory/xiRT)

For manuscripts utilizing custom algorithms or software that are central to the research but not yet described in published literature, software must be made available to editors and reviewers. We strongly encourage code deposition in a community repository (e.g. GitHub). See the Nature Research guidelines for submitting code & software for further information.

Data

Policy information about availability of data

All manuscripts must include a data availability statement. This statement should provide the following information, where applicable:

- Accession codes, unique identifiers, or web links for publicly available datasets
- A list of figures that have associated raw data
- A description of any restrictions on data availability

The mass spectrometry proteomics data have been deposited to the ProteomeXchange Consortium (http://proteomecentral.proteomexchange.org) via the jPOST partner repository69 with the dataset identifier PXD020407 and DOI 10.6019/PXD020407 [http://proteomecentral.proteomexchange.org/cgi/GetDataset?ID=PXD020407]. Raw data of the FA-Complex is available via the previously published PRIDE identifier PXD014282 [http://proteomecentral.proteomexchange.org/cgi/

GetDataset?ID=PXD014282]. Additional files and intermediate results are available via Zenodo 10.5281/zenodo.4270324 [https://zenodo.org/record/4270324]. Source data are provided with this paper. PPI data was retrieved from STRING data (https://string-db.org/, v11) and APID (http://cicblade.dep.usal.es:8080/APID/init.action, downloaded 09/2019).

Field-specific reporting

Please select the one below that is the best fit for your research. If you are not sure, read the appropriate sections before making your selection.

- [x] Life sciences
- [ ] Behavioural & social sciences
- [ ] Ecological, evolutionary & environmental sciences

For a reference copy of the document with all sections, see nature.com/documents/nr-reporting-summary-flat.pdf

Life sciences study design

All studies must disclose on these points even when the disclosure is negative.

Sample size	Sample size were not predetermined. Our rational was to choose a sample size large enough to generate a diverse collection of cross-linked peptides from many protein-protein-interactions to train a deep neural network. As shown in Fig. 2 (d,e,f,g) the collected data suffices to train accurate deep learning models.
Data exclusions	No data were exluded.
Replication	Most samples were measured once because replicate measurements would have tremendously increased the acquisition time and cost. However, note that we also successfully tested our workflow on a publicly available data set.
Randomization	Samples were acquired in a non-random order to minimize the influence of sample carry-over within the acquisition queue and so to improve any prediction from machine learning.
Blinding	Not applicable, because there is no expected observer bias.

Reporting for specific materials, systems and methods

We require information from authors about some types of materials, experimental systems and methods used in many studies. Here, indicate whether each material, system or method listed is relevant to your study. If you are not sure if a list item applies to your research, read the appropriate section before selecting a response.

Materials & experimental systems

n/a	Involved in the study	
[x]	[ ]	Antibodies
[x]	[ ]	Eukaryotic cell lines
[x]	[ ]	Palaeontology and archaeology
[x]	[ ]	Animals and other organisms
[x]	[ ]	Human research participants
[x]	[ ]	Clinical data
[x]	[ ]	Dual use research of concern

Methods

n/a	Involved in the study	
[x]	[ ]	ChIP-seq
[x]	[ ]	Flow cytometry
[x]	[ ]	MRI-based neuroimaging
